# Expression of basement membrane laminin in oral squamous cell carcinomas

**DOI:** 10.1016/S1808-8694(15)31173-3

**Published:** 2015-10-19

**Authors:** Silvia Regina de Almeida Reis, Leonardo Francisco Provedel de Souza, Verônica Ferreira de Souza, Lílian Dantas de Góes Silva, Jean Nunes dos Santos

**Affiliations:** 1 Master's degree in Dentistry, Substitute Professor of the Integrated Clinic and Propedeutics Department, Dentistry School of the Bahia Federal University; 2 Master's degree in Dentistry, Assistant Professor, Dentistry School of the Southwestern Bahia State University; 3 Bolsista do Programa de Educação Tutorial, Graduanda da Faculdade de Odontologia da Universidade Federal da Bahia; 4 Doctorate in Dentistry, Adjunct Professor of the Integrated Clinic and Propedeutics Department, Dentistry School of the Bahia Federal University; 5 Doctorate in Dentistry, Adjunct Professor of the Integrated Clinic and Propedeutics Department, Dentistry School of the Bahia Federal University

**Keywords:** carcinoma, laminin, basement membrane

## Abstract

The basement membrane is a dynamic structure that undergoes quantitative and qualitative changes during the progression of squamous cell carcinoma, which is essencially important in tumoral invasion and metastasis.

**Aim:**

This study is aimed at investigating the behavior of the basement membrane in oral squamous cell carcinomas with different malignancy scores, which were obtained through the immunohistochemical expression of the laminin, a glycoprotein present in the basement membrane.

**Study design:**

History cross-sectional cohort.

**Material and method:**

Thirty-one cases of oral squamous cell carcinoma were subjected to histological grading of malignant tumors. The immunohistochemical expression of the laminin in lesions bearing different scores of malignancy was evaluated according to intensity and integrity, using the Streptavidin-Biotin complex method.

**Results:**

We noticed significant differences in the media between intensity and continuity laminin expression in relation to different grades of malignancy.

**Conclusion:**

Different expressions of laminin, a glycoprotein present in basement membranes were evident in oral cell carcinomas within different grades of histological malignancy.

## INTRODUCTION

Cancer is surely among the major causes of death worldwide. Research has, for many years now, sought effective treatment for this disease. The single point of agreement is that all forms of treatment work best when the malignancy is diagnosed early. This statement is the basis of a ceaseless search for increasingly accurate diagnostic methods to improve treatment adequacy.

Immunohistochemical studies of basal membrane components have been shown to be effective in diagnosing and establishing the prognosis of cancer. Basal membrane components are not only an important structural barrier, but also act as a barrier against neoplastic invasion in epidermoid carcinomas, thus avoiding tumor cell dissemination.^2^

Laminin is a glycoprotein that is present in the basal membrane; it has specific actions, including an adhesion function. Tumor cells bind to laminin receptors on the basal membrane and are subsequently stimulated to produce metaloproteinases, which begins fragmentation and degradation of the membrane. These features justify immunohistochemical studies of basal membrane components that might be related to tumor invasion.

Recently, Garcia et al. (2006)^3^ investigated the immunohistochemical expression of collagen IV and laminin in samples of normal epithelium, mild and moderate dysplasias, in situ carcinomas, invasive carcinomas and metastatic nodules. The authors observed a progressive loss of continuity in the immunohistochemical expression of these proteins as tissues progressed from dysplasias to metastatic nodules; they also noted that a weak expression of markers was significant in the progression of such mouth lesions. This relation is important, as more aggressive carcinomas have an increased ability to produce enzymes that degrade basal membrane components, consequently hindering new protein synthesis.

This paper aims to investigate the immunohistochemical expression of laminin in the basal membrane of oral squamous cell carcinoma, and to correlate this expression with the histological features of malignancy.

## MATERIAL AND METHOD

The Research Ethics Commite of the Dentistry School, Bahia Federal University, approved this study; the protocol number was 011/05.

Histological studies were made of 31 biopsies of primary oral squamous cell carcinomas. None of the subjects had been treated previously with chemotherapy or radiotherapy. There were 23 males (74.2%) and eight females (27.8%). Subjects were aged between 29 and 81 years (mean age = 60 years. The tongue was the most frequent tumor site, followed by the floor of the mouth. Caucasian subjects predominated (15 cases; 48.4%), followed by brown skin color (9 cases; 29%) and black subjects (7 cases; 22.6%).

Biopsy specimens were fixated in formaldehyde 10% and paraffin blocked; 5 mm thick sections were made for morphological analysis. Slide for each case were hematoxylin-eosin (H&E) stained and examined under light microscopy to describe morphological findings. A magnification of 400X was used for the histological classification according to the malignant tumor classification system proposed by Anneroth, Batsakis & Luna (1987)^4^ (Table 1). This system requires assessing six morphological features that characterize the tumor cell population and the tumor-host relation, as follows: degree of keratinization, nuclear pleomorphism, number of mitoses, invasion pattern, invasion stage and lymphoplasmocytic infiltration. The parameter “invasion stage” was omitted as we studied incision biopsies of oral squamous cell carcinomas. Each morphological parameter was graded from 1 to 4. The end score for each case was defined as the arithmetic mean of the sum of points given to each parameter that was assessed. Results between 1.0 and 2.5 were low degrees of malignancy, which values between 2.6 and 4.0 meant a high degree of malignancy.

**Table 1:** Malignancy grading system for oral squamous cell carcinoma according to Anneroth, Batsakis and Luna (1987).

Points				
Morphological parameters	1	2	3	4
Degree of keratinization	Highly keratinized (50% of cells)	Moderately keratinized (20-50% of cells)	Minimally keratinized (2-5% of cells)	No keratinization (0-5% of cells)
Nuclear pleomorphism	Discrete (75% mature cells)	Moderate (50-75% of mature cells)	Abundant (25-50% mature cells)	Extreme (0-25% mature cells)
Number of mitoses	0 – 1	2 – 3	4 – 5	5
	Histological malignancy grade and the tumor-host relation			
Points				
Morphological parameters	1	2	3	4
Invasion pattern	Well-defined margins	Solid cords and/or islets	Small cells groups (n =15)	Marked cell dissociation (n =15)
Invasion stage	In situ carcinoma and/or unsure invasion	Invasion involving only the lamina propria	Invasion immediately below the lamina propria	Extensive and deep invasions
Lymphoplasmocytic infiltrate	Marked	Moderate	Discrete	Absent

Immunological laminin glycoprotein marking was done using the streptavidin-biotin method. Immunohistochemical testing was done on 3μm-thickness sections of the paraffin-blocked material. Paraffin was removed from these sections, which were then immersed in a TRIS solution at pH 7.4. The anti-laminin monoclonal antibody was used as the primary antibody (LAM-89, SIGMA 1:75). The following steps required an immunohistochemical marking automatized system (Autostainer Universal Staining System Dako) for incubation of the primary and secondary antibodies and the streptavidin-biotin tertiary complex. Between reactions the sections were vigorously washed in TRIS pH 7.6 and Twiin 0.5% solutions. Processing of the reaction with diaminobenzidine (DAB Plus) and Mayer's hematoxylin counter staining was done in the device. Sections of healthy oral mucosa were used as a positive control; the same tissue, after suppressing the primary antibody reaction, was used as a negative control.

Histological analysis of the immunohistochemical expression of laminin in sections of all cases of oral squamous cell carcinomas was done without prior knowledge of the histological grading of malignancy for each lesion. A semi-quantitative method was used to grade the intensity and the integrity of laminin expression in the basal membrane according to Shinohara et al.'s (1996)^2^ and Kannan et al.'s (1994)^5^ methodology. Marking intensity was graded as absent (-), discrete (+), moderate (++) and intense (+++). The integrity of basal membrane laminin was graded according to the following criteria: continuous (+++), when laminin marking was linear, with no interruptions, similar to normal epithelium; moderately continuous (++), when immune reactivity was above or equal to 50% of the basal membrane, discretely continuous (+), when less than 50% of the basal membrane was positive for the antibody or absent/nearly absent (±). Areas with intense inflammatory infiltrates were not assessed to avoid distorting the results.

A descriptive analysis was made to identify the general and specific features of the sample. Mann-Whitney's non-parametric test was used to check for significant associations between the intensity and continuity of laminin immunohistochemical expression in the high and low malignancy groups. Spearman's correlation test was used to check for the existence of a linear relation between the intensity and continuity of laminin and the malignancy grade. Statistical significance was a p-value < 0.05.

## RESULTS

Morphologically, 19 (61.2%) of 31 cases had low malignancy grades (score between 1.0 and 2.5 points), while 12 cases (38.7%) had scores equal to or above 2.6, which was indicative of high malignancy grade tumors.

**Figure 1:**
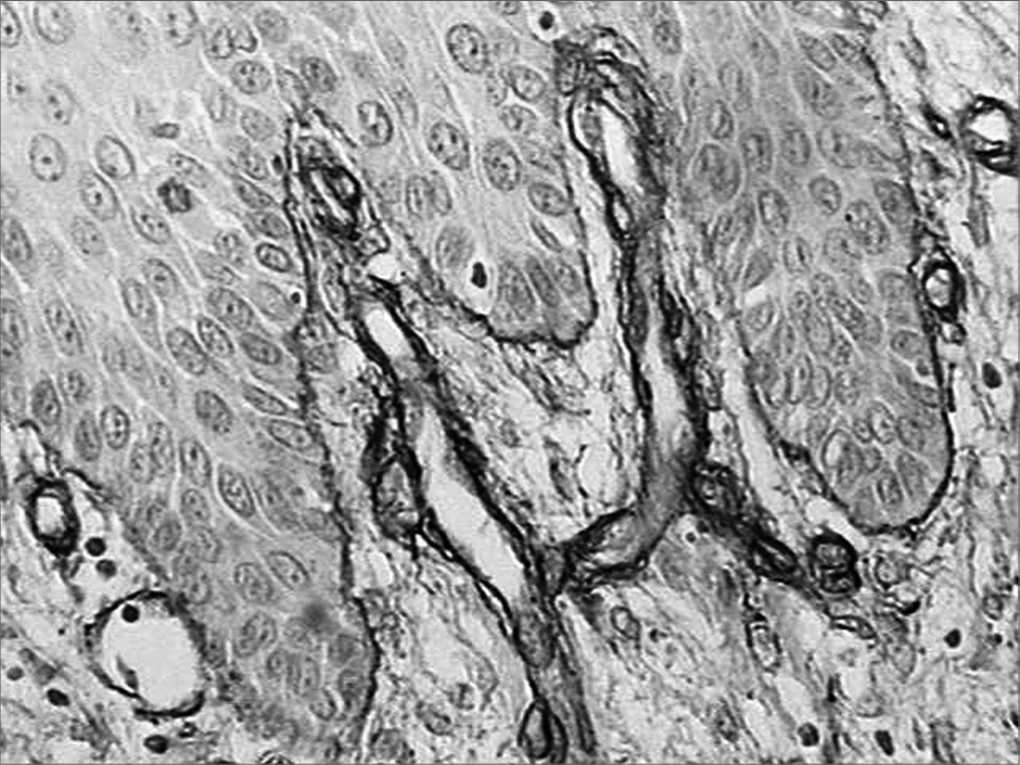
Immunohistochemical expression of laminin in the basal membrane of an oral squamous cell carcinoma showing linear, uninterrupted and continuous marking. (Streptavidin-biotin, 100X)

**Figure 2:**
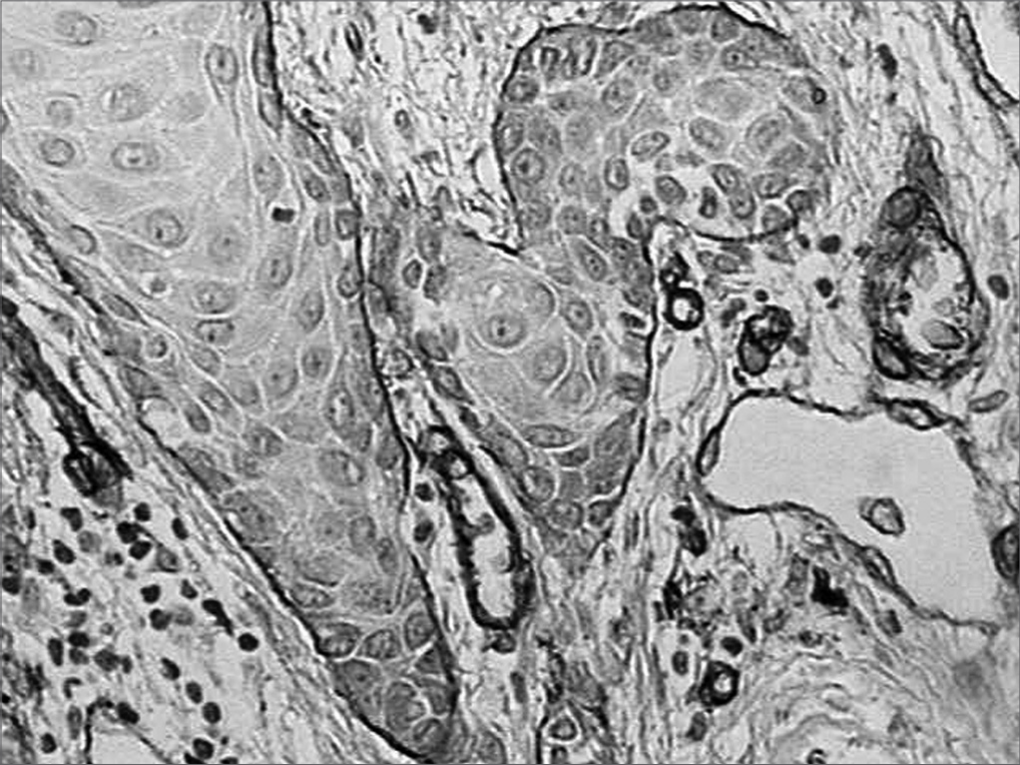
Intense immunohistochemical expression of laminin in the basal membrane of an oral squamous cell carcinoma. Note the areas with loss of continuity. (Streptavidin-biotin, 100X)

**Figure 3:**
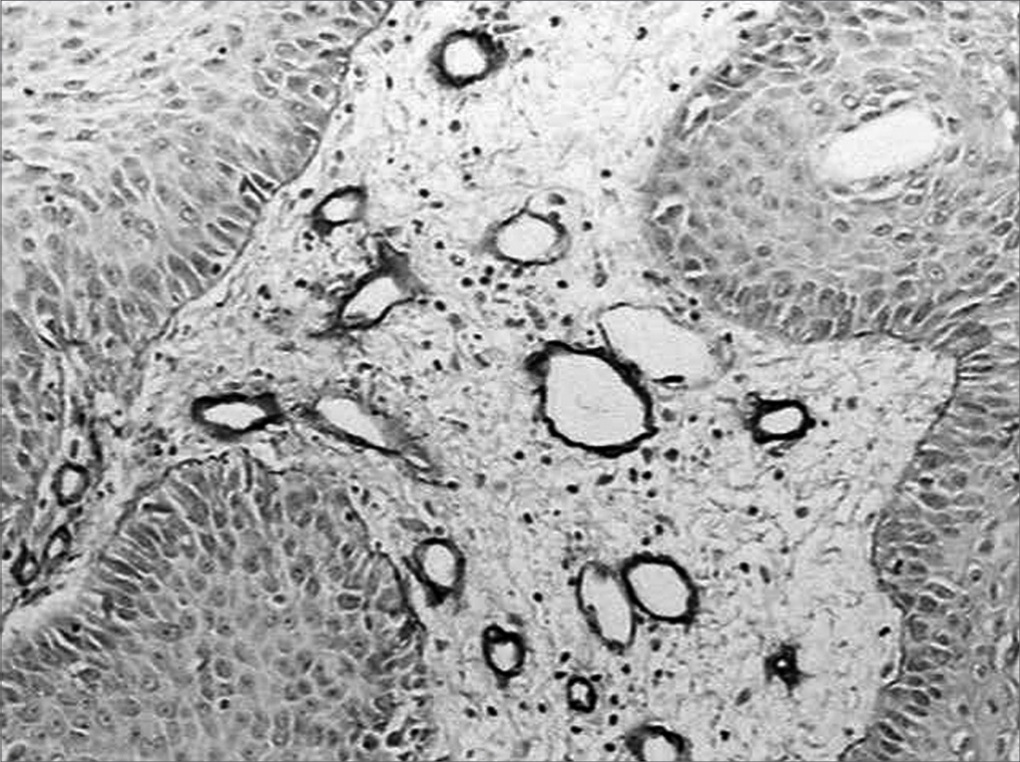
Moderate immunohistochemical expression of laminin in the basal membrane of an oral squamous cell carcinoma. (Streptavidin-biotin, 100X)

**Figure 4:**
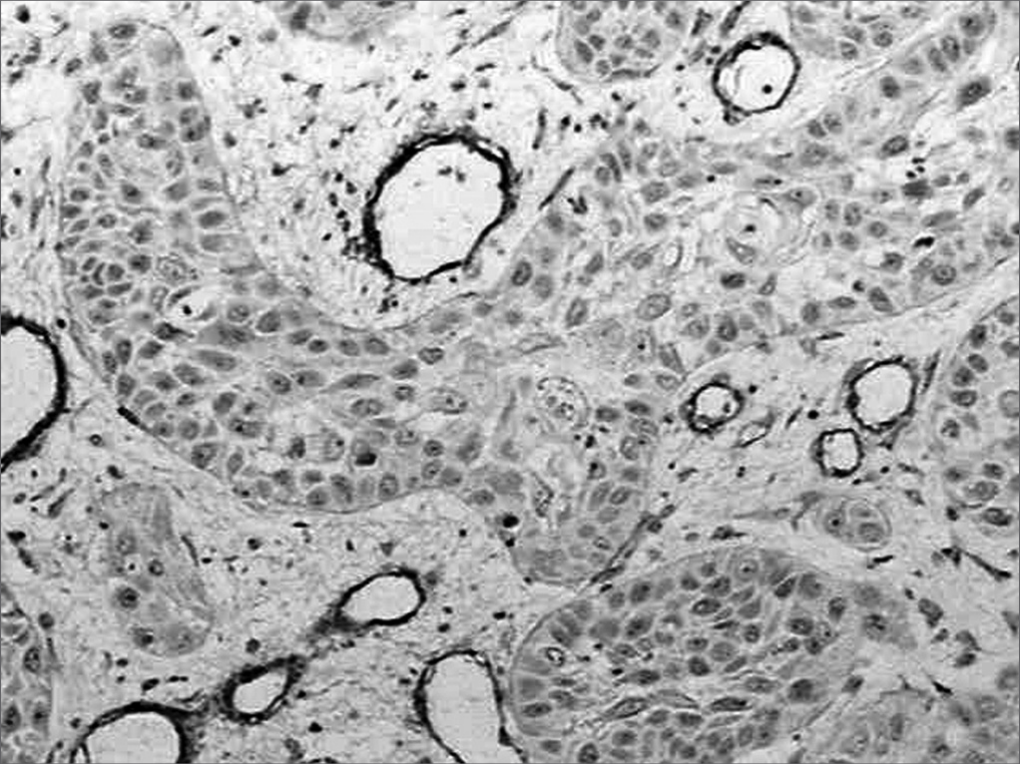
Discrete immunohistochemical expression of laminin. Note the intense marking of blood vessels in the tumor stroma. (Streptavidin-biotin, 100X)

There was intense laminin marking (+++) surrounding the borders of neoplastic cell clusters in seven low malignancy grade cases (36.8%) (Figures 1 and 2). Moderate laminin expression (++) was found in five cases (26.3%) and discrete laminin expression (+) was found in seven cases (36.8%) (Figures 3 and 4). The continuity or integrity of laminin in the basal membrane was related to the intensity of laminin expression. The continuous pattern (+++) was found in 10 cases (52.6%); 5 cases (26.3%) had moderate continuity (++), while discrete continuity (+) was seen in three 3 cases (15.7%). Near absence of continuity (+/-) of the basal membrane in this low malignancy grade group was found in a single lesion (5.2%).

**Figure 5:**
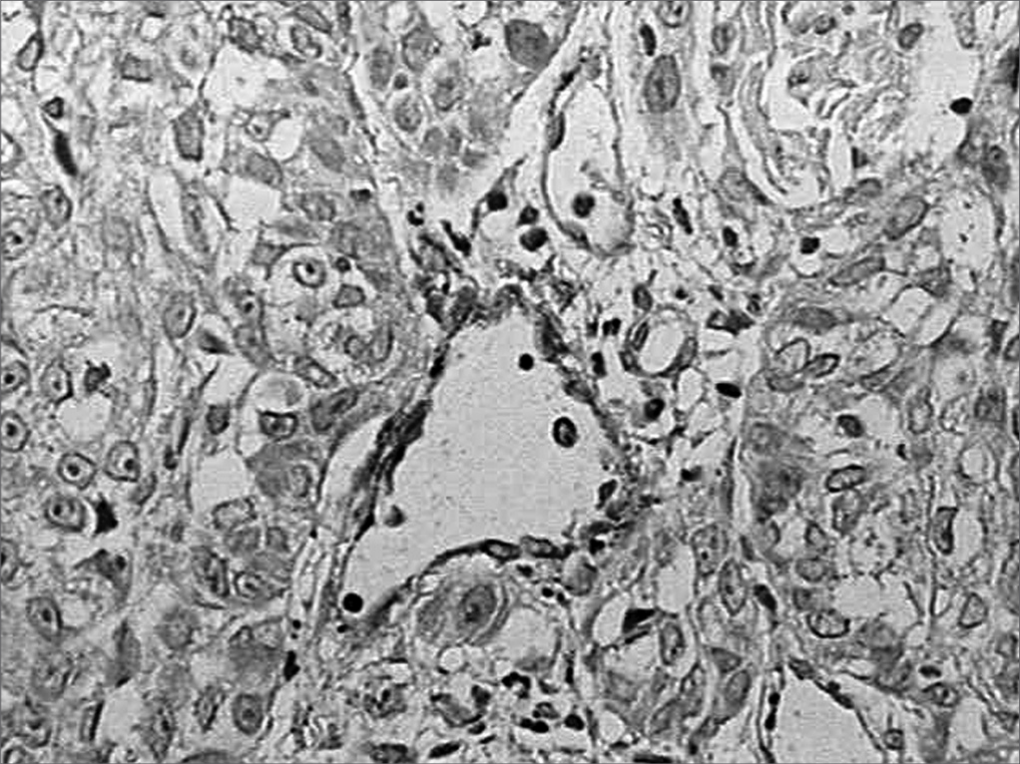
Absence of immunohistochemical expression of laminin. (Streptavidin-biotin, 100X)

Discrete laminin expression predominated in highly malignant squamous cell carcinoma cases (66.6%), while moderate expression (++) was found in two cases (16.6%); the remaining two cases (16.6%) - with higher malignancy grade lesions - there was no expression of laminin (-) (Figure 5). There were no cases of intense laminin marking (+++). Half of this group (six cases) had discrete continuity (+), characterized by less than 50% of laminin expression in the basal membrane. Moderate continuity (++) was expressed in four 4 cases (33.3%) and near total loss of continuity (+/-) was found in two cases (16.6%). No lesion showed a linear continuity (+++) pattern similar to the normal epithelial basal membrane.

There were statistically significant differences in a comparison of laminin immunohistochemical intensity and continuity mean values in low and high grade malignancies. (Table 2).

**Table 2:** Distribution of laminin marking intensity and continuity mean values in high and low malignancy grade squamous cell carcinomas.

Carcinoma	n	Intensity Mean DP +-	p	Continuity Mean DP +	p
Low Grade	19	2,0 ± 0,8	0,003*	2,26 ± 0,9	0,001*
High Grade	12	1,0 ± 0,6		1,17 ± 0,7	

* Statistically significant at 5%

Spearman correlation test demonstrated a statistically significant correlation between laminin intensity and continuity marking and the histological malignancy grade of the carcinomas seen in this study (Table 3).

**Table 3:** Spearman's matrix for correlating the variables laminin marking intensity and continuity.

Variables	Intensity	Continuity	Malignancy grade
Intensity	1	0,794**	-0,496**
Continuity	0,794**	1	-0,444*
Malignancy grade	-0,496**	-0,444*	1

** Statistically significant correlation at 1%

* Statistically significant correlation at 5%

## DISCUSSION

The extracellular matrix is composed of proteins and polysaccharides that are secreted locally and distributed in an extensive organized network associated with cell surfaces. The interface between the epithelium and the connective tissue is a matrix named the lamina or basal membrane, a thin but resistant layer that has an important role in controlling cell behavior.^6^ It is composed of, among others, type IV collagen, heparan sulphate, fibronectin, entactin and laminin;^7^ the latter is a glycoprotein that is involved in cell adhesion, cell migration, proteolytic activity, cell proliferation, and tumor and metastatic growth.^8^

The basal membrane is a dynamic, rather than a static structure, being continuously remodeled by glycoprotein rupture and synthesis (such as laminin). These processes are extremely important in inflammation and tissue repair, as the membrane becomes fragmented to allow inflammatory cell entry and exit. Such fragmentation needs to be orderly, rapidly and accurately repaired; this process does not take place if tumor cells are present. During neoplastic invasion, glycoprotein synthesis in the basal membrane is slower and disorganized, leading to permanent loss of continuity.^5^ In recognition of this feature, our analysis of oral mucosal squamous cell carcinomas for assessing laminin expression was not done in areas with intense inflammation, to avoid distorting the results.

The basal membrane is the most significant barrier for invading neoplastic cells.8 Oral squamous cell carcinoma cells have been shown to contain 32/67kDa laminin receptors; these receptors operate as an accessory integrin molecule that stabilizes tumor cells upon their adherence to laminin. After establishing this bond, the tumor begins to secrete enzymes^9^,^10^ that cause membrane rupture by destroying type IV collagen and laminin; tumor cells are then able to penetrate the connective tissue and start the invasion process.^11^,^12^ These changes in basal membrane distribution have been demonstrated by immunohistochemical techniques using anti-collagen IV and anti-laminin antibodies,^3^ the latter of which was used in this study.

The basal membrane is more than a support structure and a barrier against neoplastic cells; it is also involved in signaling during angiogenesis, since various angiogenesis-modulating molecules, growth factors and cytokines are stored in the basal membrane to be released and activated when the basal membrane is breached. Laminin degradation exposes angiogenesis-inducing sites, which is essential for neoplastic cell survival.^8^ Our study confirms this finding, since we observed an increased number of blood vessels adjacent to the neoplastic invasion front, compared to normal epithelium.

Loss of the basal membrane, evidenced by decreased expression of laminin and collagen IV, has been noted in areas where differentiation is lost,^3^,^13^,^14^ suggesting that such regions will shortly be the site of progressive neoplastic invasion into the adjacent stroma.^15^ Jiang et al. (1994)^16^ and Harada et al. (1994)^17^ have demonstrated this finding by observing lower laminin expression in metastatic tumors. In our study, of 12 high malignancy lesions, two did not express laminin, while discrete intensity marking was seen in another 8 lesions. An interesting histological finding was the absence of intense laminin marking in this group of cases. Statistically significant differences were noted by confronting intensity and continuity mean values with malignancy grades. Aznavoorian et al.'s (1993)^11^ and Nigar & Dervan's (1998)^18^ immunohistochemical studies of the basal membrane in oral squamous cell carcinomas reached similar results.

Laminin has been investigated in other lesions such as skin and salivary gland tumors^19^,^20^, ^21^, ^22^ and in colon and rectal malignant neoplasms.^9^ Some of these papers revealed that increased laminin expression was found in the basal membrane of well-differentiated lesions. Our findings were similar to these results; statistically significant evidence shows that an increased intensity of laminin expression was correlated with more differentiated lesions.

Our analysis of laminin expression and basal membrane integrity showed that no highly malignant lesions had a continuous marking pattern (that would be similar to normal epithelium). In this group, however, we found that in four lesions the basal membrane had over 50% integrity. In their papers Kumagai et al. (1994)^23^ and Bosman & Stamenkovic (2003)^24^ reached similar results, having established that loss of basal membrane components does not always occur at every instant of tumor invasion. The basal membrane that surrounds tumor cells is fragmented during active invasion stages and is rebuilt during latency stages.

Differentiated and consequently less invasive tumors showed a laminin marking expression that was very similar to normal epithelium. In our study, of 19 low malignancy grade tumors, 10 cases showed continuity in laminin expression. This finding suggests that adjacent cells to the basal membrane and tumor cells were still able to secrete laminin, confirming similar description in the literature.^7^,^8^,^12^, ^25^

Maatta et al. (2001)^1^ published the results of specific marking of various laminin isoforms, and found that human carcinomas are generally capable of synthesizing sizeable amounts of different laminin chains. It was also noted that carcinomas contained nearly all of the existing laminin chains in their basal membranes, suggesting the macromolecule deposition on the basal membrane is not totally lost during tumor invasion.^15^,^26^ This might explain moderate laminin expression in two high malignancy grade lesions in our study.

Firth & Read (1996)^15^ found a high frequency of focal laminin defects in the basal membrane of clinical stage T3 and T4 tumors. Similar results were seen in clinical stage T1 and T2 tumor.^23^ Based on this study we understand that the expression of basal membrane components is better correlated with tumor differentiation stages, rather than with their clinical grade.

## CONCLUSION

We concluded that there was decreased immunohistochemical expression of laminin in the basal membrane of high malignancy grade oral squamous cell carcinomas. We suggest that this structural change may affect basal membrane dynamism and favor tumor invasion.
